# Changing Geographic Patterns and Risk Factors for Avian Influenza A(H7N9) Infections in Humans, China

**DOI:** 10.3201/eid2401.171393

**Published:** 2018-01

**Authors:** Jean Artois, Hui Jiang, Xiling Wang, Ying Qin, Morgan Pearcy, Shengjie Lai, Yujing Shi, Juanjuan Zhang, Zhibin Peng, Jiandong Zheng, Yangni He, Madhur S. Dhingra, Sophie von Dobschuetz, Fusheng Guo, Vincent Martin, Wantanee Kalpravidh, Filip Claes, Timothy Robinson, Simon I. Hay, Xiangming Xiao, Luzhao Feng, Marius Gilbert, Hongjie Yu

**Affiliations:** Université Libre de Bruxelles, Brussels, Belgium (J. Artois, M. Pearcy, M.S. Dhingra, M. Gilbert);; Chinese Center for Disease Control and Prevention, Beijing, China (H. Jiang, Y. Qin, S. Lai, Y. Shi, Z. Peng, J. Zheng, L. Feng, H. Yu);; Fudan University School of Public Health, Key Laboratory of Public Health Safety, Ministry of Education, Shanghai, China (X. Wang, S. Lai, J. Zhang, Y. He, X. Xiao, H. Yu);; Fonds National de la Recherche Scientifique, Brussels, Belgium (M. Pearcy, M. Gilbert); University of Southampton, Southampton, UK (S. Lai);; Food and Agriculture Organization of the United Nations, Rome, Italy (M.S. Dhingra, S. von Dobschuetz, T. Robinson);; Food and Agriculture Organization of the United Nations Regional Office for Asia and the Pacific, Bangkok, Thailand (F. Guo, W. Kalpravidh, F. Claes);; Food and Agriculture Organization of the United Nations China Office, Beijing (V. Martin); University of Washington, Seattle, Washington, USA (S.I. Hay);; University of Oxford, Oxford, UK (S.I. Hay); University of Oklahoma, Norman, Oklahoma, USA (X. Xiao)

**Keywords:** Influenza in humans, geographic mapping, influenza A virus, H7N9 subtype, poultry, influenza, China, viruses

## Abstract

The fifth epidemic wave of avian influenza A(H7N9) virus in China during 2016–2017 demonstrated a geographic range expansion and caused more human cases than any previous wave. The factors that may explain the recent range expansion and surge in incidence remain unknown. We investigated the effect of anthropogenic, poultry, and wetland variables on all epidemic waves. Poultry predictor variables became much more important in the last 2 epidemic waves than they were previously, supporting the assumption of much wider H7N9 transmission in the chicken reservoir. We show that the future range expansion of H7N9 to northern China may increase the risk of H7N9 epidemic peaks coinciding in time and space with those of seasonal influenza, leading to a higher risk of reassortments than before, although the risk is still low so far.

The third and fourth epidemic waves of avian influenza A(H7N9) human infections in China showed an apparent reduction in incidence compared to the spring 2013 and winter 2013–14 epidemic waves. However, during the winter of 2016–17, the incidence rose, growing to levels never observed before and reaffirming concerns of a pandemic threat posed by the H7N9 virus (*1*–*3*). Since 2013, more than 1,520 human cases of H7N9 virus infection have been reported, mostly located in eastern China, with a case-fatality rate ranging from 30% to 40% (*4*–*6*).

The H7N9 virus that caused the first epidemic wave in March 2013 originated from multiple reassortment events of avian influenza viruses from domestic poultry and wild birds (*7*). Mainly restricted to the Yangtze River Delta in eastern China, including urban areas of Shanghai, Jiangsu, and Zhejiang Provinces, in the first wave, the spatial range of H7N9 human cases increased during the second wave along the coast into Guangdong Province in southern China (*8*). Over time, phylogeographic inference suggested that H7N9 had become established in separate parts of China during the second and third waves, reassorting with local avian influenza viruses (*9*,*10*).

Humans are not a natural reservoir, but occasional spillover hosts of H7N9 human cases act as indicators, presumably reflecting the circulation of H7N9 in poultry (*10*), and are an effective way of studying the spatial distribution of H7N9 virus. Surveillance in poultry is difficult, as the virus has so far had a low pathogenicity in chickens (*11*,*12*), and the absence of clinical signs means that active and targeted sampling is needed. This difficulty has made the characterization of the spatial distribution of the virus reservoir inconclusive, although that may change in the future because of the recent evolution of a highly pathogenic strain of H7N9 (*13**–**15*).

In this study, we considered 3 sets of factors that may influence the spatial variation in H7N9 incidence. The first set of spatial risk variables, termed anthropogenic variables, included the distribution of live-poultry markets (LPMs) and human population density. Visits to LPMs are the main known risk factor for H7N9 human infection (*16*–*18*), and LPMs represent a key interface between humans and poultry. At a higher level, LPM networks may also support the spread and persistence of H7N9 virus through the network of LPMs and poultry farms linked by trade (*19*). In previous studies, we showed that a high density of LPMs in some specific areas could regionally increase the risk for H7N9 infection at the market level (*20*), which translates into higher risk at the county level, as observed in several studies (*21*–*23*). Human population density was included as a surrogate for surveillance bias and to account for any anthropogenic transmission mechanisms.

During the fifth wave, outbreaks in poultry farms started to be reported in higher numbers, so we included a second set of predictor variables, termed poultry, including the density of chickens and ducks, as these may regionally influence the risk of H7N9 virus transmission to humans. From 69% to 80% of H7N9 human patients in the 5 epidemic waves reported exposure to live poultry before infection, including LPM (52%–60%) and backyard poultry (13%–40%); these figures remained fairly stable with time (*1*). Although most of those exposures may correspond to LPM visits, other opportunities for contact with poultry along the production and value chain also exist. For example, poultry workers in Beijing were shown to be at a higher risk for H7N9 infection than the remaining population of the city (*24*). Poultry may become a reservoir when the circulation of avian influenza viruses through the production and value chains cannot be prevented; poultry-related variables were found to be key predictors of H7N9 risk in several previously published studies (*20*,*23*,*25*,*26*).

In addition, to account for the distribution and abundance of wild birds, we included 2 indicator variables of proximity to and abundance of water and wetlands. Although the most conservative hypothesis remains that human infections are linked to the circulation of H7N9 in domestic poultry with exposure in LPMs, it cannot be assumed that wild birds do not play any role in transmission. The virus precursors of the H7N9 virus in China were found in a wide variety of bird species, both wild and domestic (*7*); avian influenza viruses circulating in wild birds represent a gene pool that may recombine with H7N9 viruses and allow better adaptation and persistence. There is little information on the wild host specificity of H7N9, and data on the distribution of wild bird species are generally coarse, with populations varying strongly according to the season.

We studied the spatial variation of H7N9 incidence in the human population during the 5 epidemic waves in relation to these 3 sets of spatial risk factors. More specifically, we compared the association between these spatial factors and H7N9 infections across the 5 epidemic waves, to investigate the spatial distribution of repeated recurrences and the year-to-year variation in predictability of H7N9 infections.

## Materials and Methods

### Data

#### H7N9 Human Cases and Seasonal Influenza

We analyzed all confirmed H7N9 human cases during February 19, 2013–August 9, 2017. We collated information on laboratory-confirmed H7N9 human cases by collecting data from the World Health Organization (WHO) Monthly Risk Assessment Summary report, websites of the national and provincial Health and Family Planning Commission of China, FluTrackers (http://flutrackers.com), HealthMap (http://www.healthmap.org/en/), and avian influenza reports from the Centre of Health Protection of Hong Kong. When information was inconsistent, we used the WHO report as the primary source. A detailed description of case definitions, surveillance for identification of cases, and laboratory testing for H7N9 virus have been provided elsewhere (*4*,*27*,*28*). For each case, the information about place of residence and date of onset of symptoms was used and 6.5 days were subtracted from the date of onset of symptoms to estimate the dates of first contact with the virus, as estimated elsewhere (*29*). To compare the seasonality of H7N9 human cases with that of human seasonal influenza A in space and time, we extracted influenza sentinel surveillance data for January 2013–March 2017 from Influenza Weekly Reports, managed by the Chinese National Influenza Centre (http://www.chinaivdc.cn/cnic/zyzx/lgzb/). More information on the sentinel network supporting these data can be found in Yu et al. (*30*).

#### Live Poultry Markets and Permanent Closure Measures

We assembled a database recording the locations of 8,943 retail and wholesale LPMs from multiple sources. In addition, we compiled a database recording the market closure measures implemented since the first wave, with the start and end date of each measure. Both databases are described in the Technical Appendix.

#### Spatial Predictor Variables

The first set of predictor variables included the LPM density (LPM/km^2^) and human population density (persons/km^2^). Some counties do not have LPMs but their inhabitants may easily go to LPMs in neighboring counties. LPMs may also act at a higher level by providing a network of markets through which the disease could spread and persist. The LPM density was computed by means of a Gaussian smoothing kernel function with the optimal bandwidth found by Gilbert et al. (*20*). To account for closure of LPMs, the data on permanent market closures were used to remove the permanently closed markets from the full LPM database before the Gaussian smoothing, resulting in a different LPM density distribution for each epidemic wave. Human population density was taken from the 2010 census (http://www.stats.gov.cn/tjsj/pcsj/rkpc/6rp/indexch.htm).

The second set of predictor variables included chicken and domestic duck densities from a new dataset we produced using the Gridded Livestock of the World methodology applied to an extensively improved dataset we compiled using the 2010 reference year (*31*,*32*). Because a high correlation was noted between duck and chicken densities at the county level, and to reduce colinearity and to facilitate the interpretation of the results, we combined these variables to give a poultry density layer (chickens + ducks/km^2^) and the chicken-to-duck ratio (chicken density/duck density).

The last set of predictor variables was indicative of water bird habitat. This included the distance to the largest lakes and reservoirs (km), measuring the distance between the county centroids and the nearest lakes (area ≥50 km^2^) or reservoirs (storage capacity ≥0.5 km^3^) (*33*), and the proportion (%) of the county covered by wetlands, according to the hybrid wetland map for China (*34*).

#### Analyses

The analyses involved the development of Poisson boosted regression tree (BRT) models to predict the daily incidence rate of H7N9 virus in the human population as a function of 6 predictor variables. (A description of the BRT models and a list of model parameters is provided in the Technical Appendix.) The models were developed using the number of human cases as the dependent variable, with an offset term corresponding to the product of human population by the duration of the epidemic. The duration of each epidemic was defined as the period separating the 5th from the 95th percentile of the days of onset of illness in each wave. One model per epidemic wave was built to compare the effect of predictor variables and to assess the predictive capacity from one wave to another. The contribution of each predictor variable to the model was quantified by its relative contribution (RC), a measure of its overall importance in the model (*35*), and by its partial dependence plots, or BRT profiles, which provide a graphical description of its effect on the daily incidence rate after accounting for the average effects of all other predictor variables in the model (*36*). We tested the presence of spatial autocorrelation in the model residuals using spline correlograms (*37*) and we used the approach of Crase et al. (*38*) when autocorrelation was present in the model residuals. To evaluate the models for their capacity to discriminate between the presence and the absence of human cases at the county level, we converted the predicted daily incidence rate into a probability of having >1 human case in the county using a binomial model. Finally, we replicated the analysis with generalized linear models because BRT models do not explicitly allow the formal testing of the significance of individual risk factors.

## Results

**Figure 1:**
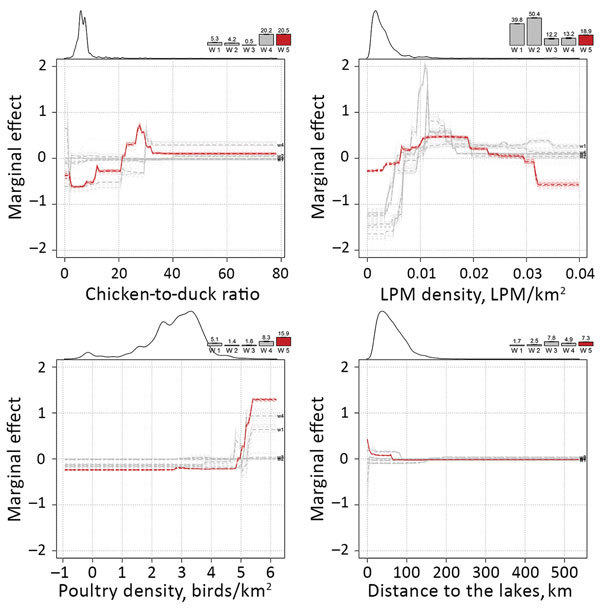
Marginal effect plots of the top 4 predictor variables on the predicted incidence rate of influenza A(H7N9) in China. Change in relative contribution over time is indicated by the bars on the top of each plot, showing the increasing relative contribution of the poultry predictor variables. The smoothed line on the top left part of each plot is indicative of the distribution of each variable.

Table 1 presents the RC of the predictor variable in the different epidemic waves. The RCs of anthropogenic predictor variables were high initially but decreased strongly after the third epidemic wave (w1 = 41.66%; w2 = 50.99%; w3 = 39.93%; w4 = 17.31%; w5 = 21.52%). In parallel, the RC of poultry predictor variables increased and was greatest in the last epidemic wave (w1 = 10.39%; w2 = 5.57%; w3 = 2.12%; w4 = 28.53%; w5 = 36.37%). In this last epidemic wave, the most noteworthy predictor variables were, in decreasing order of RC values, the chicken-to-duck ratio (20.49%), the LPM density (18.41%), the poultry density (15.88%), and the distance to open lakes and reservoirs (7.31%). Figure 1 presents the BRT profiles of these 4 predictor variables in the different epidemic waves (the other profiles are provided in Technical Appendix Figure 1). The chicken-to-duck ratio had a notable RC only in waves 4 and 5, when it showed a positive association with incidence up to a ratio of ≈30. The LPM density profile of wave 5 also showed a positive association, leveling off at a density of 0.01, showing a profile that was relatively similar to those of the other epidemic waves. Wave 5, in contrast to previous epidemic waves, tended to associate lower incidence with the highest LPM densities (>0.03). The poultry density profile changed gradually over time, with an increasing RC, and the incidence rate in wave 5 is predicted to increase strongly in counties with a high density of poultry (>60,000 birds/km2). Finally, the profile of the distance to lakes showed a decreasing association in the range 0–100 km.

**Table 1:** Relative contribution of the different Poisson BRT models across 5 epidemic waves of influenza A(H7N9), China*

Model	Relative contribution ± SD, %				
	Wave 1	Wave 2	Wave 3	Wave 4	Wave 5
Anthropogenic†	41.66	50.99	39.93	17.31	21.52
LPM density	39.81 ± 0.24	50.43 ± 0.42	12.22 ± 0.78	13.24 ± 0.78	18.91 ± 0.12
Human population density	1.85 ± 0.14	0.56 ± 0.03	27.71 ± 0.49	4.07 ± 0.22	2.61 ± 0.04
Poultry†	10.39	5.57	2.12	28.53	36.37
Chicken-to-duck ratio	5.33 ± 0.18	4.18 ± 0.06	0.54 ± 0.06	20.23 ± 0.3	20.49 ± 0.18
Poultry density	5.06 ± 0.14	1.39 ± 0.04	1.58 ± 0.13	8.3 ± 0.36	15.88 ± 0.08
Water habitat†	2.18	3.6	9.29	5.68	8.48
Proportion of wetlands	0.49 ± 0.02	1.13 ± 0.06	1.51 ± 0.1	0.74 ± 0.07	1.17 ± 0.03
Distance to lakes	1.69 ± 0.05	2.47 ± 0.11	7.78 ± 0.23	4.94 ± 0.19	7.31 ± 0.1
Autoregressive term	45.77 ± 0.27	39.84 ± 0.32	48.65 ± 0.65	48.49 ± 1.33	33.62 ± 0.17
					

*BRT, boosted regression tree; LPM, live poultry market.

†Sum of relative contribution for both categories.

The assessment of the BRT models’ goodness of fit is presented in Table 2. With the exception of the fourth epidemic wave, the predictability of the models was moderate, with cross-validation correlation coefficients ranging from 0.42 to 0.55. For the presence/absence term, the models had a good discriminatory capacity, with areas under the curve (AUCs) ranging from 0.77 to 0.92, but this capacity decreased over the years (w1 = 0.92; w2 = 0.85; w3 = 0.83; w4 = 0.86; w5 = 0.77). This finding implies that it was easier to predict the presence or absence of a human case (good discrimination capacity and AUC values) than it was to predict the number of cases (moderate predictability and correlation coefficients). The discriminatory capacity was maintained from wave to wave, with a lower overall AUC in wave 5 (Table 3). The results obtained with the same risk factors and dependent variable from the generalized linear models (Technical Appendix) show a similar pattern, with poultry variables becoming more apparent after the fourth epidemic wave.

**Table 2:** Goodness-of-fit metrics of the Poisson BRT models across 5 epidemic waves of influenza A(H7N9), China*

Wave	Pearson correlation coefficient ± SD				AUC ± SD	
	Training	Training, auto	Cross-validation		Training	Training, auto
1	0.793 ± 0.011	0.553 ± 0.002	0.487 ± 0.014		0.924 ± 0.001	0.907 ± 0.001
2	0.749 ± 0.004	0.345 ± 0.008	0.55 ± 0.014		0.849 ± 0.001	0.848 ± 0
3	0.588 ± 0.01	0.496 ± 0.003	0.424 ± 0.013		0.833 ± 0.002	0.811 ± 0.001
4	0.423 ± 0.005	0.292 ± 0.007	0.258 ± 0.009		0.855 ± 0.001	0.833 ± 0.001
5	0.586 ± 0.001	0.539 ± 0.001	0.446 ± 0.009		0.773 ± 0	0.75 ± 0

*AUC, area under the curve; BRT, boosted regression tree.

**Table 3:** Cross-predictability of the BRT models trained with the different epidemic waves of influenza A(H7N9), China, applied to the others, as measured by the area under the curve*

Predictions	Applied to				
	Wave 1	Wave 2	Wave 3	Wave 4	Wave 5
Wave 1	0.91	0.81	0.78	0.84	0.79
Wave 2	NA	0.85	0.78	0.83	0.76
Wave 3	NA	NA	0.82	0.82	0.74
Wave 4	NA	NA	NA	0.83	0.75
Wave 5	NA	NA	NA	NA	0.76

*BRT, boosted regression tree; NA, not applicable.

**Figure 2:**
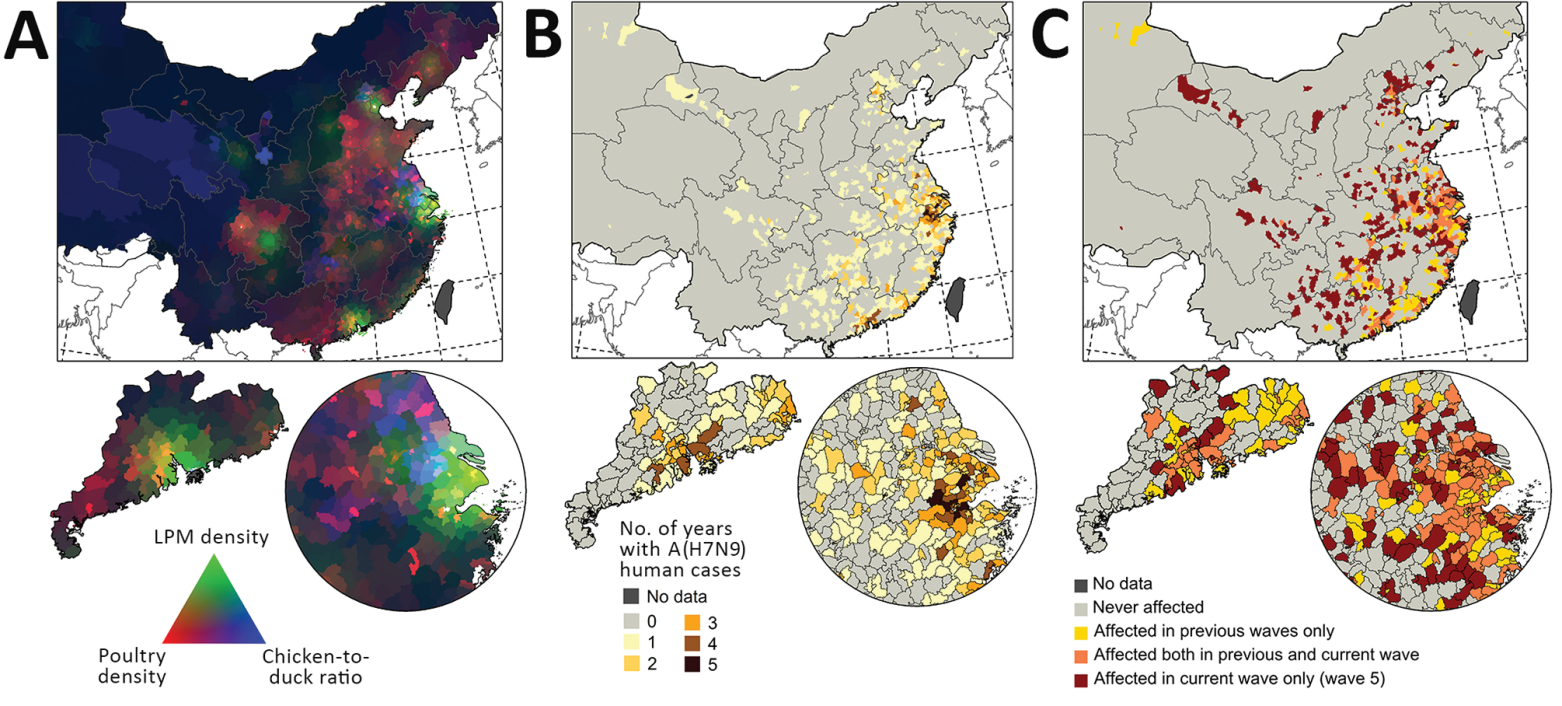
Distribution of predictor variables and influenza A(H7N9) infections in China, with 3 geographic extents: smallest extent around the location of human cases (top), Guangdong Province (bottom left), and Yangtze River Delta (bottom right). A) Visualization of poultry density (red), live-poultry market density (green), and chicken-to-duck ratio (blue). Dark areas correspond to low values and light areas to high values in all 3 predictors. B) Number of years with >1 human case per county. C) Distribution of the fifth wave of human infections compared with previous waves.

**Figure 3:**
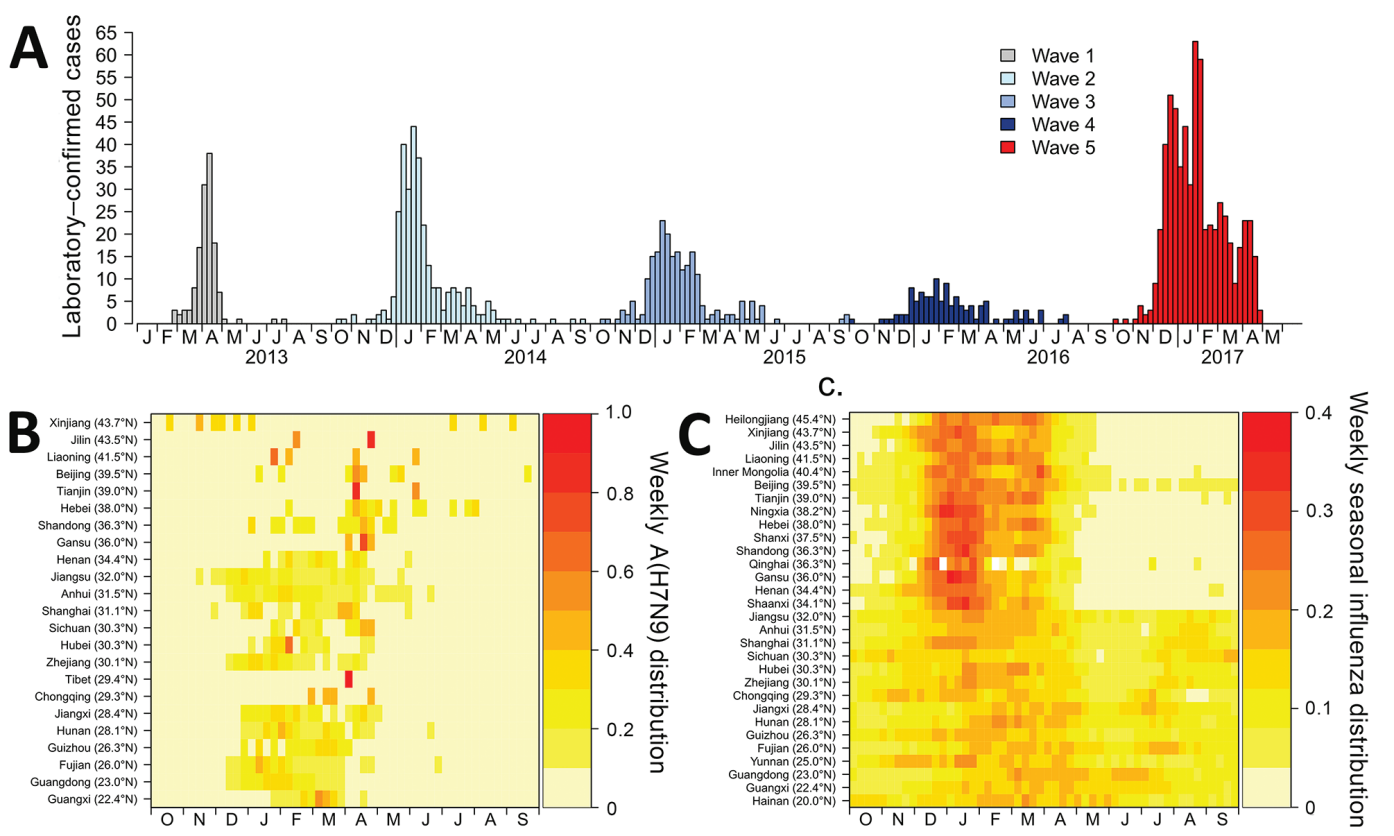
Seasonality of influenza A(H7N9) infections in comparison to seasonal influenza, by month, China, 2013–2017. A) Epidemic curve for H7N9. B) Seasonality for H7N9. C) Seasonality for seasonal influenza.

Figure 2 shows the distribution of the top 3 predictor variables (LPM density, poultry density, and chicken-to-duck ratio) in relation to the distribution of the human cases, distinguishing those from epidemic waves. The RGB (red/green/blue) composite plot (Figure 2, panel A) highlights areas in which all 3 predictor variables were high and where H7N9 persisted over time (Figure 2, panel B). A large area to the east of Taihu Lake on the urban areas of Wuxi, Suzhou, and Shanghai had high LPM densities and included several small hotspots of high poultry density. The RGB composite plot shows 3 additional areas with high LPM densities and high poultry densities: Guangdong Province, the Tianjin and Beijing urban areas; and the Chongqing urban area. These areas visually correspond to areas of high H7N9 recurrence in Figure 2, panel B, which contrasts counties with repeated recurrences from those with sporadic infections. Figure 2, panel C illustrates that the spatial pattern of wave 5 showed a marked geographic expansion from these previous hotspots of persistence, with 279 counties reporting H7N9 for the first time (66.11% of the total number of counties infected in wave 5). It is also apparent why LPM density was a less powerful predictor variable in wave 5 than in previous waves, as these newly infected counties no longer correspond to the green areas depicted in Figure 2, panel A. The heat maps shown in Figure 3 show that the majority of H7N9 human cases occurred around February and March (Figure 3, panel B), with a latitudinal gradient. The seasonality of common influenza A infection is different throughout China (Figure 3, panel C), with the provinces north of 34.1 degrees showing a much stronger annual winter seasonality of infection than do the more southerly provinces, where most cases occurring during December– February. A comparison of Figure 3, panels B and C, shows that the peaks of H7N9 and seasonal influenza A have so far not coincided strongly in space and time. However, a geographic range expansion of H7N9 infections into the northern provinces, retaining the current seasonality, would bring the H7N9 and seasonal influenza A incidence peaks toward each other in both space and time.

## Discussion

The results of our spatial models demonstrate a major shift over time from anthropogenic toward poultry predictor variables linked to H7N9 human cases, apparent in wave 4 and confirmed in wave 5. This shift was evident in both BRT and generalized linear models. More specifically, the predictive power of poultry variables increased over time and was greatest in the last epidemic, pointing to areas with high chicken densities and high chicken-to-duck ratios. A recent study on H7N9 human cases showed an increase in periurban and rural cases in the fifth wave and a comparatively higher number of cases among middle-aged persons (*1*). However, apart from the overall increase in cases, the study did not suggest any other major epidemiologic differences, and other authors have made similar observations when comparing waves 1–4 (*1*,*5*,*8*). Our results do not contradict the observation of a higher number of human cases in periurban and rural areas, because high poultry production regions are typically located in periurban and rural settings, but they strongly support the hypothesis that the H7N9 virus may have spread in the chicken reservoir much more extensively during the last 2 epidemic waves than was previously the case, with a particularly marked geographic range expansion in the last epidemic wave. This observation, based on human cases, can be linked to the emergence of HPAI H7N9 that was reported early in 2017 in southern China (*13*). Recently published results showed that human cases of HPAI H7N9 were already found beyond Guangdong, in Hunan and Guangxi Provinces in early 2017 (*15*). In parallel, a comparatively higher number of reports of H7N9-positive samples was found in chicken farms this year in comparison with previous epidemic waves, including reports of HPAI H7N9 in northern China, in Tianjin (*39*). The precise role of the gain in pathogenicity on the range expansion of H7N9 remains unclear, as do the main mechanisms of transmission along the poultry production and value chain networks. However, the fact that such a range expansion took place in parallel with the emergence of a highly pathogenic variant seems unlikely to be coincidental.

It should be noted that the measure of predictor weights in the model, the RC, is relative, so that the sum of RCs equals 1. If, therefore, the poultry variables become better predictors of H7N9 incidence in humans, the RC of other variables must decrease, even if their effect on the predicted incidence remains fairly constant. The contribution of LPMs may have remained high, but its combination with increasing transmission along the poultry production and value chains may be responsible for the geographic range expansion and higher incidence observed during the fifth wave.

Although some of the highest incidences of H7N9 were observed along Taihu Lake, the predictive capacity of variables associated with water birds had a much lower influence in the models than did the anthropogenic and poultry variables. Many interfaces combining wetlands, intensive poultry farming, and rice paddy fields are present in southeastern China and may have played a role in the initial emergence of the H7N9 virus in the Shanghai area (*40*). As the virus spread in the following epidemic wave, however, the contribution of wild birds to overall disease circulation may be fairly low, which is reflected by the low relative contribution of the water bird habitat proxy variables.

The predictive capacity of the incidence models was only moderate, as these spatial models did not account for the variability in incidence linked to market closure measures. In contrast, the predictions of presence/absence were generally better because presence cannot be influenced by market closure measures; such measures usually followed human cases rather than precedinged them, and few counties implemented market closure measures in the absence of human cases.

This moderate predictive capacity may also relate to some limitations of the study. There may be an underreporting of milder symptomatic infections (*30*), and the effect and geographic distribution of this bias is unknown. Another aspect is that the poultry dataset underlying our analyses is of uneven quality, with better and more detailed data in the east than in western parts of the country, as shown by Artois et al. (*26*). Finally, although all efforts were made to compile the most comprehensive LPM dataset possible, many LPMs may have opened and closed, including illegal ones, further adding to model uncertainty. Finally, we were not able to integrate poultry movement and trade data (legal or illegal) into this analysis because of the lack of centralized data; this may be a line of investigation for the future.

The geographic range expansion and increase in incidence of human cases in the fifth wave of H7N9 brings serious human health concerns. First, repeated human infection by avian influenza viruses increases the chances of virus recombination, mutation, or both, leading to human-to-human transmission. Second, the provinces affected by earlier H7N9 epidemic waves do not have a strong seasonal influenza A peak in January and February (*30*) that matches the peak of H7N9 cases (Figure 3). However, if the H7N9 virus continues to expand its range northward, in areas with a strong influenza A peak in January and February, there will be a higher chance of local coincidence of peaks of incidence between human cases of H7N9 and seasonal influenza A virus. This change may enhance the chances of coinfections that could lead to the emergence of reassortants with the capacity to transmit easily between humans. Third, the extent of the geographic range of the expansion is not yet fully known; in the absence of new measures, it may spread further within China and internationally through poultry value chains.

**Technical Appendix.** Additional information about the methods and databases used in study of 5 waves of influenza A(H7N9) infection in China.
