# Supplementary material for: Changing Geographic Patterns and Risk Factors for Avian Influenza A(H7N9) Infections in Humans, China
Source: Emerg Infect Dis. 2018 Jan;24(1):87–94. doi: 10.3201/eid2401.171393 (PMC5749478; doi:10.3201/eid2401.171393)
Supplement: Supplementary file 1 — Technical Appendix. Additional information about the methods and databases used in study of 5 waves of influenza A(H7N9) infection in China. [file 17-1393-Techapp-s1.pdf]

Article DOI: http://dx.doi.org/10.3201/eid2401.171393

Changing Geographic Patterns and Risk Factors for Avian Influenza A(H7N9) Infections in Humans, China

Technical Appendix

Live Poultry Markets

We compiled a database recording the locations of live-poultry markets (LPMs) and types of market closure measures implemented since the first wave, with start and end dates. The database was initially described elsewhere (*1*) and assembled by combining data from the official website of the Ministry of Agriculture of China and agricultural bureaus at the province and prefecture levels, a database of points of interest from the official gazetteer issued by the National Administration of Surveying, Mapping, and Geoinformation, and several unpublished sources obtained through data mining, Internet searches, and direct contacts with provincial agricultural bureaus. Our database recorded the type, starting date, end date, and location of market closure measures that were implemented since the first wave. A total of 38 types of measures over different time periods were implemented in response to the H7N9 human infections and the market closures were implemented at the county or district level. We reclassified the 38 types of measures into 4 categories according to the closure measures, as follows (cleaning and disinfections measures were not analyzed): LPMs that were permanently closed (permanent); LPMs that were closed for 1 or 2 days with a recursive repetition of the closing, for which the period between measures could be a week or a month (recursive); LPMs that were temporarily closed for a short period, ranging from 1 day to 1 week (short period); and LPMs that were temporarily closed for a duration ranging between 1 week and the full duration of the epidemic (long period). A count of closing measures along the epidemic waves is presented in Technical Appendix Table 1. Only data on permanent market closures were used to update a yearly distribution of LPM locations used in this study, ranging from 1 to 32 permanent closures per epidemic waves.

Boosted Regression Tree Models

The analyses involved the development of Poisson boosted regression tree (BRT) model were discussed previously (*2*,*3*). Poisson regression allows for predicting a variable with a count response, such as the number of human cases per county. Poisson models handle exposure variables (offset terms) by using simple algebra to change the dependent variable from a rate (count/exposure) into a count. BRTs are machine learning methods and combine 2 algorithms: regression trees and boosting. They belong to the family of species distribution models because they can deal with abundance and absence/presence data. BRT models generate a large number of regression trees, fitted in a stepwise manner, for optimizing the predictive probability of occurrence based on predictor variable values. A possible disadvantage to BRT is that it does not have the facility to assess the statistical significance of individual effect variables; for this reason, the analysis was repeated with classical generalized linear models. However, BRT models have been shown to produce accurate predictions of the distribution of avian influenza diseases (*1*,*4*,*5*) and are capable of fitting models that account for nonlinear effects, and for interactions between predictor variables. They also ensure that the effects of extreme outliers and the inclusion of irrelevant predictors are not a source of bias for model predictions (*6*). We developed each epidemic wave model using a 4-fold cross-validation procedure (*3*) as a key step to control and limit model overfitting, which is frequently associated with machine learning methods. Finally, the analysis was bootstrapped initially with 30 independent BRT runs for a total of 120 cross-validations (30 runs, 4-fold) per wave to account for variations in data splitting for the cross-validation. The choice of n = 30 resulted from a trade-off between processing time and the convergence of the mean, controlled after the initial runs with the standard deviation of the model metrics. BRT models were run with the following parameters: a tree complexity of 2, an initial number of trees set at 200, a learning rate of 0.003, and a step size of 50 trees.

We converted the predicted incidence rate into a probability of having at least 1 human case in the county by using a Binomial distribution, as follows: $P(X > 0) = 1 – (1 – p)^{nd}$ where nd is the population multiplied by the length of the epidemic in days and p is the incidence rate predicted by the Poisson BRT model

Additional Analysis

To check whether the distribution of human cases of the H7N9 virus in China is influenced by environmental factors, we formulated Poisson generalized linear models (GLMs) to explain the daily incidence rate (DIR) compared with human population density, LPM density, poultry density, chicken-to-duck ratio, distance to water, and the proportion of water in the county. One GLM per epidemic wave was fitted to be able to compare the effect of predictor variables between waves. The presence of spatial autocorrelation in the GLMs residuals was tested and taken into account using the same approach adopted for BRT models. The procedure is described in the main text of this article. For all analysis of variance, the effect of the predictor variables were tested and computed using a type I sum of squares procedure. In that procedure, the variance explained by the predictor variables is tested sequentially, so the predictor variables must be ordered thoughtfully. The confounding variables were added first in the models: the autoregressive terms to catch fully the spatial structures, followed by the human density variables for some surveillance and reporting biases. Then, the remaining predictor variables were incorporated in GLMs following the order of appearance and importance of these variables in the introduction. The assessment of the GLM goodness of fit is presented in Technical Appendix Table 2 and the analysis of variance tables to test the effect of predictor variables is given in Technical Appendix Table 3. Some of the GLM Poisson models are overdispersed with dispersion parameters exceeding 1 (1.37 for the epidemic wave 5; see Technical Appendix Table 2). Thus, the effects of predictor variables were computed under Poisson distribution hypotheses and quasi-Poisson distribution.

References

1. Gilbert M, Golding N, Zhou H, Wint GRW, Robinson TP, Tatem AJ, et al. Predicting the risk of avian influenza A H7N9 infection in live-poultry markets across Asia. Nat Commun. 2014;5:4116. http://dx.doi.org/10.1038/ncomms5116

2. Friedman JH. Greedy function approximation: a gradient boosting machine. Ann Stat. 2001;29:1189–232. http://dx.doi.org/10.1214/aos/1013203451

3. Elith J, Leathwick JR, Hastie T. A working guide to boosted regression trees. J Anim Ecol. 2008;77:802–13. http://dx.doi.org/10.1111/j.1365-2656.2008.01390.x

4. Martin V, Pfeiffer DU, Zhou X, Xiao X, Prosser DJ, Guo F, et al. Spatial distribution and risk factors of highly pathogenic avian influenza (HPAI) H5N1 in China. PLoS Pathog. 2011;7:e1001308. http://dx.doi.org/10.1371/journal.ppat.1001308

5. Dhingra MS, Artois J, Robinson TP, Linard C, Chaiban C, Xenarios I, et al. Global mapping of highly pathogenic avian influenza H5N1 and H5Nx clade 2.3.4.4 viruses with spatial cross-validation. eLife. 2016;5:e19571. http://dx.doi.org/10.7554/eLife.19571

6. Friedman JH, Meulman JJ. Multiple additive regression trees with application in epidemiology. Stat Med. 2003;22:1365–81. http://dx.doi.org/10.1002/sim.1501

Technical Appendix Table 1. Number of live poultry market closure measures following waves of influenza A(H7N9), China

Type of closure	Wave 1	Wave 2	Wave 3	Wave 4	Wave 5
Permanent	32	27	13	17	1
Recursive	92	59	76	18	229
Short	7	139	14	24	243
Long	137	102	163	46	326

Technical Appendix Table 2. Goodness of fit metrics of the GLMs across the different epidemic waves of influenza A(H7N9), China*

	Pearson correlation coefficient		AUC		Dispersion parameter
Wave	Training	Training (auto)	Training	Training (auto)	(quasi-Poisson)
Wave 1	0.487	0.504	0.899	0.903	0.912
Wave 2	0.413	0.396	0.813	0.813	1.192
Wave 3	0.426	0.411	0.804	0.802	1.221
Wave 4	0.206	0.205	0.834	0.835	0.723
Wave 5	0.360	0.365	0.747	0.746	1.335

*AUC, area under the curve; GLM, generalized linear model

Technical Appendix Table 3. Analysis of deviance table of generalized linear models for 5 waves of influenza A(H7N9) infections, China*

Wave	Coefficient	Df	Residual deviance	Explained deviance	p-value Poisson	p-value quasi-Poisson	% of deviance explained
Wave 1							
NULL	NA	NA	790.470	NA	NA	NA	NA
Autoregressive term	0.088	1	648.710	141.760	<0.001	<0.001	NA
Human population density	0.451	1	541.629	107.081	<0.001	<0.001	48.041
LPM density	0.293	1	447.000	94.629	<0.001	<0.001	42.455
Poultry density	0.338	1	442.877	4.124	0.042	0.033	1.85
Chicken-to-duck ratio	0.044	1	440.735	2.142	0.143	0.125	0.961
Proportion of wetland	0.033	1	440.062	0.672	0.412	0.391	0.302
Distance to water	–0.702	1	425.816	14.246	<0.001	<0.001	6.391
Wave 2							
NULL	NA	NA	1384.268	NA	NA	NA	NA
Autoregressive term	0.080	1	1130.313	253.956	<0.001	<0.001	NA
Human population density	0.291	1	1045.046	85.267	<0.001	<0.001	46.537
LPM density	0.148	1	1017.617	27.428	<0.001	<0.001	14.97
Poultry density	0.289	1	1010.821	6.796	0.009	0.017	3.709
Chicken-to-duck ratio	0.137	1	1004.635	6.186	0.013	0.023	3.376

Wave	Coefficient	Df	Residual deviance	Explained deviance	p-value Poisson	p-value quasi-Poisson	% of deviance explained
Proportion of wetland	0.166	1	965.868	38.767	<0.001	<0.001	21.158
Distance to water	0.368	1	947.087	18.781	<0.001	<0.001	10.25
Wave 3							
NULL	NA	NA	992.823	NA	NA	NA	NA
Autoregressive Term	0.121	1	867.395	125.428	<0.001	<0.001	NA
Human population density	0.757	1	764.591	102.803	<0.001	<0.001	76.172
LPM density	0.114	1	757.073	7.519	0.006	0.013	5.571
Poultry density	0.081	1	757.052	0.021	0.884	0.895	0.016
Chicken-to-duck ratio	0.101	1	756.313	0.738	0.390	0.437	0.547
Proportion of wetland	0.065	1	752.081	4.232	0.040	0.063	3.136
Distance to water	0.430	1	732.432	19.649	<0.001	<0.001	14.559
Wave 4							
NULL	NA	NA	657.843	NA	NA	NA	NA
Autoregressive Term	0.113	1	564.970	92.872	<0.001	<0.001	NA
Human population density	0.447	1	529.735	35.235	<0.001	<0.001	51.694
LPM density	0.119	1	518.771	10.964	0.001	<0.001	16.086
Poultry density	0.133	1	517.896	0.875	0.350	0.271	1.283
Chicken-to-duck ratio	0.199	1	497.660	20.236	<0.001	<0.001	29.689
Proportion of wetland	0.057	1	496.837	0.823	0.364	0.286	1.208
Distance to water	0.022	1	496.810	0.027	0.871	0.848	0.039
Wave 5							
NULL	NA	NA	2364.321	NA	NA	NA	NA
Autoregressive Term	0.106	1	1983.576	380.744	<0.001	<0.001	NA
Human population density	0.044	1	1951.345	32.231	<0.001	<0.001	38.707
LPM density	0.039	1	1943.637	7.708	0.005	0.016	9.257
Poultry density	0.229	1	1926.609	17.028	<0.001	<0.001	20.449
Chicken-to-duck ratio	0.107	1	1903.768	22.841	<0.001	<0.001	27.43
Proportion of wetland	0.025	1	1902.765	1.003	0.317	0.386	1.205
Distance to water	–0.079	1	1900.307	2.457	0.117	0.175	2.951

*Df, degrees of freedom ; LPM, live poultry market; NA, not applicable.

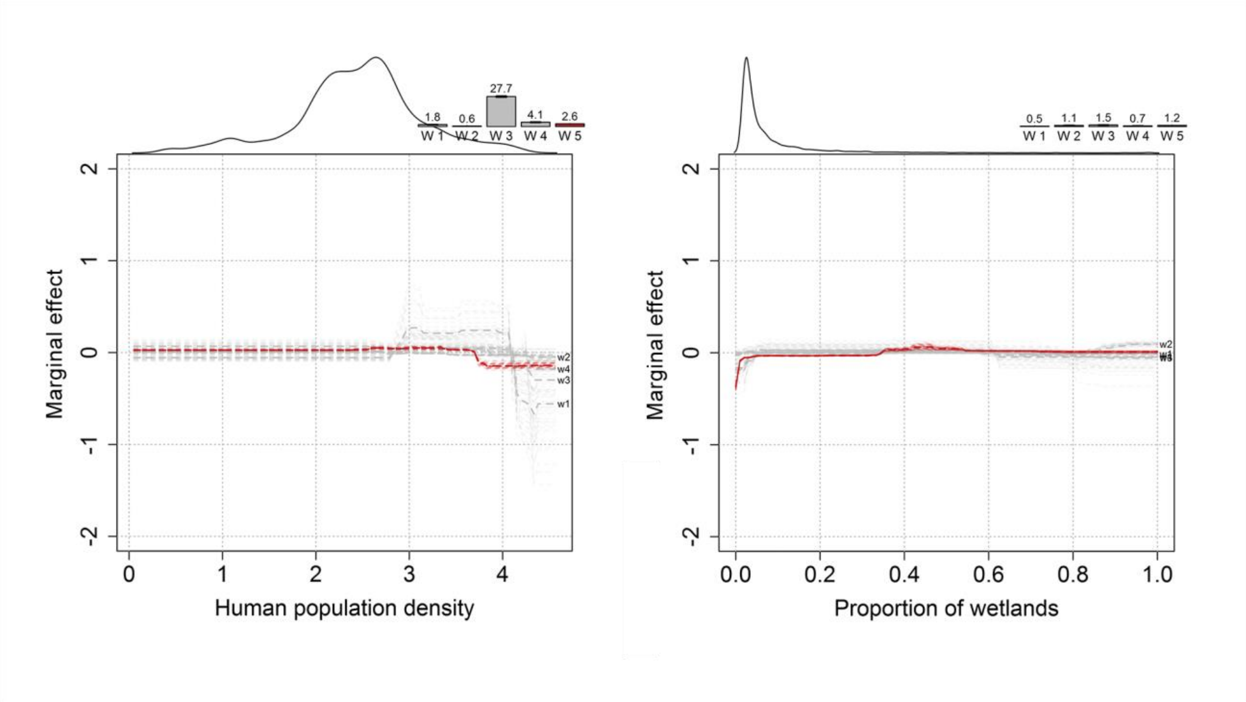

Technical Appendix Figure. Marginal effect plots of the "human density" and "proportion of wetlands" predictor variables on the predicted incidence rate, with the change in relative contribution over time indicated by the bars on the top of each plot, showing the increasing relative contribution of the poultry predictor variables. The smoothed line on the top left part of each plot is indicative of the distribution of each variable.
